# Evaluation of the protective effect of salidroside on arsenic-induced cardiac dysfunction in rats: Protective effect of Sal on arsenic-induced cardiac dysfunction

**DOI:** 10.3724/abbs.2023252

**Published:** 2023-10-30

**Authors:** Ruimeng Tian, Jing Cheng, Yanna Wang, Chuncui Chen, Lei Huang, Caiyun Zhang, Yifei Zhou, Shanshan Dong, Guilin Lu, Wenjuan Qin

**Affiliations:** Department of Ultrasonography the First Affiliated Hospital Shihezi University Shihezi 832008 China

Arsenic is a natural metal found in nature. Excessive intake of arsenic in the human body can lead to acute and chronic arsenic poisoning and even induce various diseases, such as cancers [1]. According to statistics, approximately 200 million people worldwide are exposed to the toxic effects of arsenic [2]. Previous studies showed that in the case of arsenic poisoning, arsenic could induce cells to produce excessive reactive oxygen species (ROS) through oxidative stress pathways, promote inflammatory reactions, and cause myocardial cell damage, leading to dysfunction of the heart [ 3, 4]. Salidroside (Sal) has been proven to have various pharmacological activities, such as anti-inflammatory, antioxidant, anticancer, antimetabolic disorders, and neuroprotective effects [5]. In recent years, the cardiovascular protective effect of Sal has received attention and has been used to treat various cardiovascular diseases [6].

In the present study, we hypothesized that the damaging effects of arsenic on the myocardium and the protective effects of Sal might be related to myocardial blood perfusion and endothelial cell function. Therefore, we established arsenic poisoning models and Sal intervention models. On the basis of structural and functional changes in cardiomyocytes, we further verified the protective effects of Sal on myocardial perfusion and endothelial cell function in arsenic-poisoned rats.

Forty male specific pathogen-free (SPF) grade Sprague-Dawley (SD) rats weighing 180‒200 g (provided by Shandong Experimental Animal Center, Jinan, China; licence: SCXK-2020-0005) were selected. After one week of adaptive feeding, they were randomly assigned into 5 groups (8 in each group, *n*=8): control group (Con group), negative control group (NC group), arsenic exposure group (SA group), low-dose salidroside group (Sal-L group), and high-dose salidroside group (Sal-H group). Sodium arsenite (SA; 10 mg/kg/day; Sigma Aldrich, St Louis, USA) was added to the drinking water of the SA, Sal-L, and Sal-H groups for 30 days, and the Con and NC groups drank distilled water. Subsequently, the NC group, Sal-L group, and Sal-H group were administered with 30 mg/kg/day, 15 mg/kg/day, and 30 mg/kg/day salidroside (99% purity), respectively, for 21 days. The Con group and SA group were gavaged with physiological saline for 21 days. After rat models were established in each group, myocardial contrast echocardiography (MCE) was performed using an EPIQ7C ultrasound instrument (Philips, Eindhoven, Netherlands) and SonoVue contrast agent (Bracco, Italy). The image was imported into QLAB analysis software, automatically fitting the curve to draw the time intensity curve (TIC) and obtaining the area under the curve (AUC), peak intensity (PI), water in slope (WIS), and the product of the two (WIS×PI). Blood was collected from the inner canthus vein to measure serum myocardial enzyme levels. Then, the rats were euthanized to obtain myocardial samples, and anti-PECAM-1/CD31 antibody (A01513-1; Boster, Wuhan, China) was used for immunofluorescence staining to detect the expression of CD31. The morphological changes in myocardial cells were examined by electron microscopy. Finally, myocardial homogenates were prepared to measure the levels of inflammatory factor markers and oxide-related enzymes in the myocardium. All experiments were performed in accordance with the guidelines and procedures for Experimental Animals approved by the Experimental Animal Ethics Committee of the First Affiliated Hospital of Shihezi University.

MCE can use the spatial distribution changes of microbubbles in blood vessels to intuitively reflect the myocardial perfusion status and objectively evaluate myocardial ischemia and myocardial segmental motion abnormalities [7]. In this study, the WIS and WIS×PI of Con group rats were significantly higher than those of the SA group ( *P*<0.05; Table 1), indicating that arsenic poisoning can cause damage to myocardial perfusion function in rats. However, in rats treated with Sal, the above parameters in the Sal-H and Sal-L groups were higher than those in the SA group in a dose-dependent manner ( *P*<0.05; Table 1). There was no significant difference in any parameters between the Con group and the NC group ( *P*>0.05; Table 1), indicating that Sal has a restorative effect on the damage to myocardial blood supply capacity caused by arsenic poisoning in rats.

**Table:** **Table 1** MCE parameters in rats from each group

	Control	NC	SA	Sal-L	Sal-H	*F*	*P*
PI (dB)	127.13±6.82	128.37±5.39	36.96±8.83*	72.88±9.71* ^,#^	85.40±15.86* ^,#,△^	111.74	0.001
WIS (dB/s)	29.63±6.19	28.33±6.60	6.14±2.35*	12.90±5.47* ^,#^	19.62±5.53* ^,#,△^	25.10	0.001
WIS×PI (dB ^2^/s)	3756.22±737.29	3616.53±741.08	221.83±84.73*	902.75±254.81* ^,#^	1612.90±329.21* ^,#,△^	74.79	0.001
AUC	2853.38±296.13	2890.25±229.31	670.00±159.76*	1037.00±170.17* ^,#^	1313.75±174.28* ^,#,△^	185.63	0.001

PI: peak intensity, representing myocardial blood volume; WIS: wash-in slope, representing myocardial perfusion velocity; WIS×PI represents myocardial perfusion blood flow; AUC: area under the curve. * *P*<0.05 compared with the control group; ^#^ *P*<0.05 compared with the SA group; and ^△^ *P*<0.05 compared with the Sal-L group.

**Figure 1:**
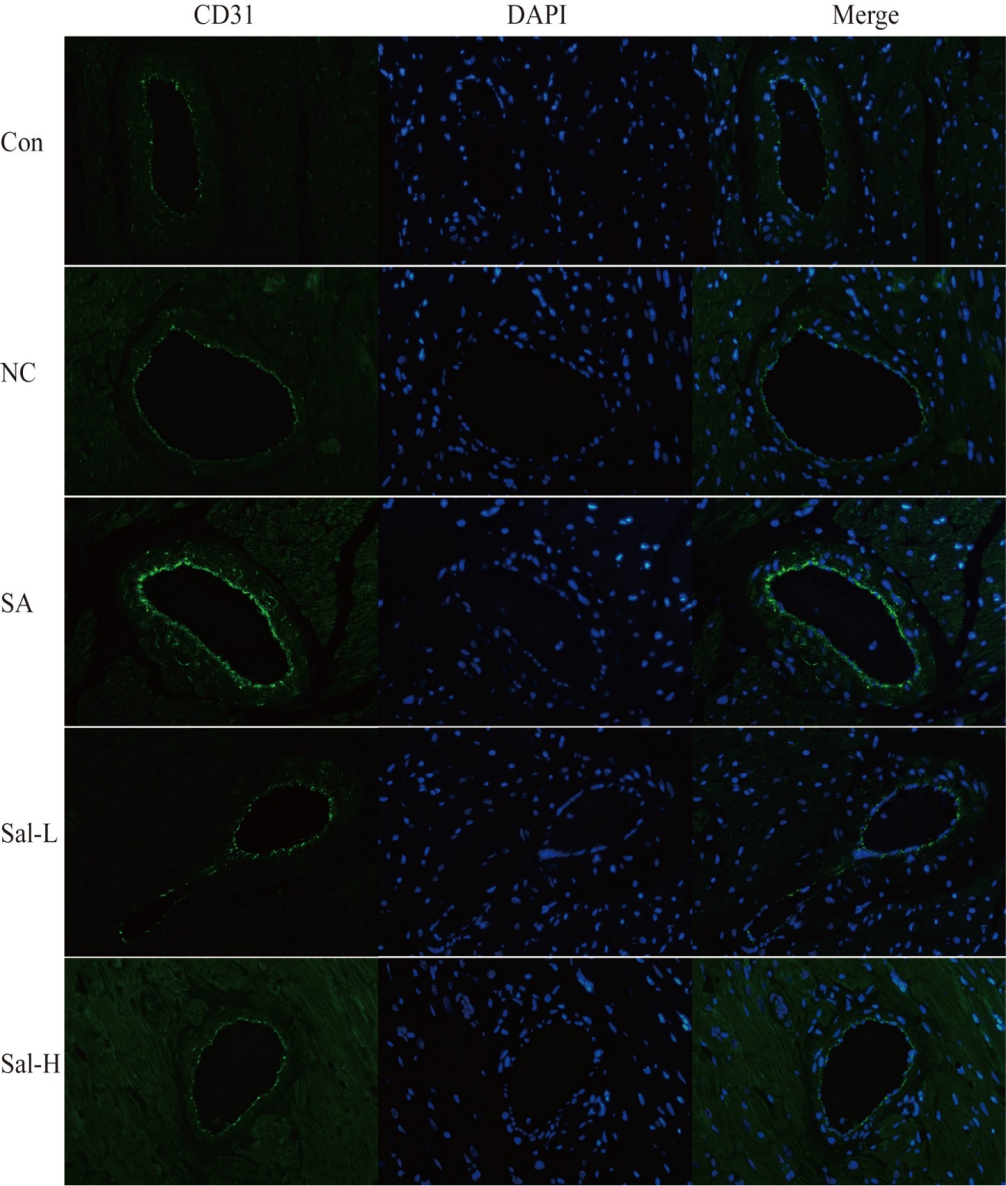
Figure 1 Immunofluorescence staining of myocardial tissues collected from each group CD31 (green) and DNA (blue). Magnification, ×400.

**Figure 2:**
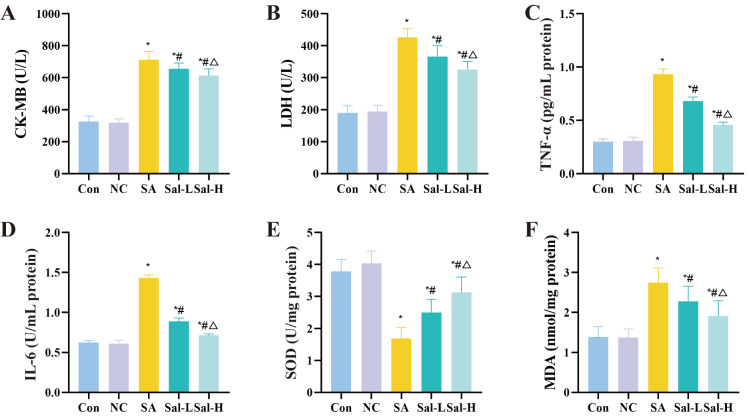
Figure 2 Myocardial CK-MB, LDH, TNF-α, IL-6, SOD and MDA levels in rats from each group (A) CK-MB. (B) LDH. (C) TNF-α. (D) IL-6. (E) SOD. (F) MDA. CK-MB: creatine kinase, LDH: lactate dehydrogenase, TNF-α: tumor necrosis factor α, IL-6: interleukin 6, SOD: superoxide dismutase, MDA: malonic dialdehyde. *P<0.05 compared with the Con group; #P<0.05 compared with the SA group; and △P<0.05 compared with the Sal-L group. Con, control.

To further evaluate the protective effect of Sal on myocardial function in arsenic-poisoned rats, platelet endothelial cell adhesion molecule (CD31) immunofluorescence staining was performed on myocardial tissue. CD31 is a transmembrane immunoglobulin-like glycoprotein expressed on the surface of white blood cells, endothelial cells, and platelets [8]. Immunohistochemical staining was used to detect the expression of CD31 in 39 rats ( Figure 1). Blue fluorescence represents the cell nucleus, while green fluorescence represents CD31 expressed by the cell. After exposure to arsenic, a large number of blue nuclei were observed in the myocardial tissue of rats, resulting in infiltration of endothelial cells, inflammatory cells, fibroblasts, etc. At the same time, the absorbance ratio of CD31 was significantly increased. After treatment with Sal, the number of infiltrating cells was decreased, and the absorbance of CD31 was also decreased. The absorbance of the Sal-H group was lower than that of the Sal-L group, and the difference was statistically significant ( *P*<0.05; Figure 2A). However, there was no clear statistical significance in the absorbance difference between the Con group and the NC group ( *P*>0.05). These results indicate that after entering the body, Sal not only damages the endothelial cells of myocardial capillaries but also promotes the infiltration of inflammatory cells into myocardial tissue. Sal can promote the recovery of damaged endothelial cells. Based on this, we speculate that Sal has a unique protective effect on vascular endothelial cells, and combined with previous MCE results, this protective effect may be one of the mechanisms that promote the recovery of blood flow perfusion in myocardial tissue damaged by arsenic poisoning.

Serum was collected from rats, and cardiac muscle enzyme levels were detected. A portion of myocardial tissue was extracted, and myocardial homogenate was made to measure the levels of peroxide-related enzymes and inflammatory factors. The levels of serum inflammatory factor markers and myocardial enzymes were detected. Correspondingly, compared with those in the Con group rats, the levels of CK-MB, LDH, TNF-α, IL-6 and MDA of the SA group, the Sal-L group, and the Sal-H group rats were increased, while SOD level was decreased. The most significant changes were observed in the SA group of rats, followed by the Sal-L group and the Sal-H group ( *P*<0.05; Figure 2 and Table 2). However, there was no significant difference between the Con and NC groups ( *P*>0.05).

**Table:** **Table 2** Biochemical indicators and CD31 absorbance in rats from each group

	Control	NC	SA	Sal-L	Sal-H	*F*	*P*
CK-MB (U/L)	327.25±32.87	318.50±24.20	712.57±51.54*	655.25±37.19* ^,#^	614.62±41.20* ^,#,△^	190.05	0.001
LDH (U/L)	189.62±22.68	193.75±19.60	426.28±27.29*	366.50±33.67* ^,#^	325.00±26.07* ^,#,△^	122.97	0.001
TNF-α (pg/mL protein)	0.30±0.03	0.30±0.04	0.93±0.05*	0.68±0.04* ^,#^	0.46±0.03* ^,#,△^	438.44	0.001
IL-6 (pg/mL protein)	0.62±0.02	0.61±0.04	1.43±0.04*	0.89±0.04* ^,#^	0.71±0.02* ^,#,△^	766.90	0.001
SOD (U/mg prorein)	3.78±0.37	4.03±0.38	1.68±0.34*	2.49±0.42* ^,#^	3.13±0.48* ^,#,△^	42.28	0.001
MDA (nmol/mg protein)	1.39±0.26	1.35±0.21	2.74±0.37*	2.28±0.38* ^,#^	1.91±0.38* ^,#,△^	24.37	0.001
CD31 (AU)	0.61±0.03	0.60±0.35	1.21±0.04*	0.89±0.04* ^,#^	0.81±0.22* ^,#,△^	250.84	0.001

CK-MB: creatine kinase, LDH: lactate dehydrogenase, TNF-α: tumor necrosis factor α, IL-6: interleukin 6, SOD: superoxide dismutase, MDA: malonic dialdehyde. * *P*<0.05 compared with the control group; ^#^ *P*<0.05 compared with the SA group; and ^△^ *P*<0.05 compared with the Sal-L group.

**Figure 3:**
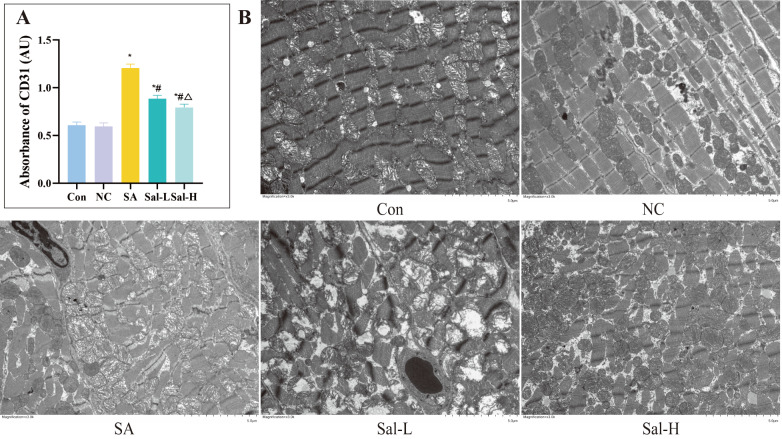
Figure 3 Myocardial CD31 level and electron microscopy of myocardial tissues collected from each group (A) Absorbance values of CD31 immunofluorescence of rats in each group. (B) Representative images of the ultrastructure of the myocardium of rats in each group (Magnification, ×3000). *P<0.05 compared with the Con group; #P<0.05 compared with the SA group; and △P<0.05 compared with the Sal-L group. Con, control.

To further verify the above experimental results, the myocardial cells of each group of rats were examined by electron microscopy. The results showed that the microstructure of myocardial fibers in the Con and NC groups was intact and neatly arranged, and the morphology of mitochondria and nuclei was normal. In contrast, myocardial fibers in the SA group underwent myofilament lysis and breakage, myofilament bundle separation, irregular Z-line arrangement, unclear boundaries between the bright and dark bands, and even some myocardial cells undergoing nuclear pyknosis and fragmentation ( Figure 3A). In addition, the normal cristae structure of mitochondria is reduced or even lost, with swelling or even rupture of volume, membrane rupture, and matrix outflow. A vesicular structure with focal cavitation was also observed. However, these damage changes, including muscle filament swelling and breakage, as well as the clarity and integrity of the nucleus and mitochondria, were improved to varying degrees in the Sal-L group and the Sal-H group. The degree of improvement in the Sal-H group was significantly better than that in the Sal-L group ( Figure 3B).

In summary, this study confirmed the destructive effects of arsenic poisoning on endothelial cells and cardiomyocytes in rats by examining blood perfusion status and myocardial endothelial cell function. Sal can counteract the arsenic damage to the rat myocardium by inhibiting oxidative stress and inflammatory response and protecting endothelial cells, providing a new research approach for the treatment of cardiac dysfunction caused by arsenic poisoning. In the future, the does-dependent cardioprotective effect of Sal will be explored.
